# *Lentilactobacillus buchneri* domination during the fermentation of Japanese traditional fermented fish (funazushi)

**DOI:** 10.1002/fsn3.3002

**Published:** 2022-07-27

**Authors:** Koichi Tanabe, Masaki Monguchi, Ryoga Inoue, Rio Zamami, Ryo Nakanishi, Ayano Manabe, Kaho Oe, Noriko Komatsuzaki, Jun Shima

**Affiliations:** ^1^ Faculty of Agriculture Ryukoku University Otsu Japan; ^2^ Microbial Resource Center for Fermentation and Brewing Ryukoku University Otsu Japan; ^3^ Graduate School of Agriculture Ryukoku University Otsu Japan; ^4^ Department of Human Nutrition Seitoku University Chiba Japan

**Keywords:** environmental stress tolerance, fermented foods, Funazushi, lactic acid bacteria, *Lentilactobacillus buchneri*

## Abstract

Funazushi is a Japanese traditional fermented fish made with boiled rice without the addition of microbial starter cultures. Isolates from various commercial funazushi products, as identified by 16S rDNA sequences, suggested that *Lentilactobacillus buchneri* strains are major lactic acid bacteria. Based on an analysis of the putative CRISPR (clustered regularly interspaced short palindromic repeat) region, the genetic diversity of *L. buchneri* strains was examined. The data suggested that the diversity of *L. buchneri* strains depended on the factories at which funazushi was produced. An analysis of samples during fermentation indicated that the transition of microbes occurred, and *L. buchneri* was the dominant species. To determine the factors associated with domination, bacteriocin production and environmental stress tolerance, including NaCl and organic acid (lactate and acetate) tolerance, were evaluated. *L. buchneri* isolates did not produce bacteriocin. Although the isolates did not exhibit NaCl tolerance, they displayed higher lactate tolerance than other lactic acid bacteria isolated during funazushi fermentation. Based on reports that *L. buchneri* can convert lactate to acetate, the previous and present results suggested that lactate tolerance and lactate conversion in *L. buchneri* could explain its domination in funazushi. Our study presented a model for the domination mechanisms of specific microbes in fermented foods by spontaneous fermentation.

## Graphical abstract

This study investigated the major lactic acid bacteria in funazushi, Japanese traditional fermented fish made with boiled rice without the addition of microbial starter cultures. An analysis of samples during fermentation of funazushi indicated that the transition of microbes occurred, and *Lentilactobacillus buchneri* became the dominant species. The tolerance to lactate in *L. buchneri* could explain its domination in the fermented food.

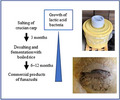

## 1 INTRODUCTION

**FIGURE 1:**
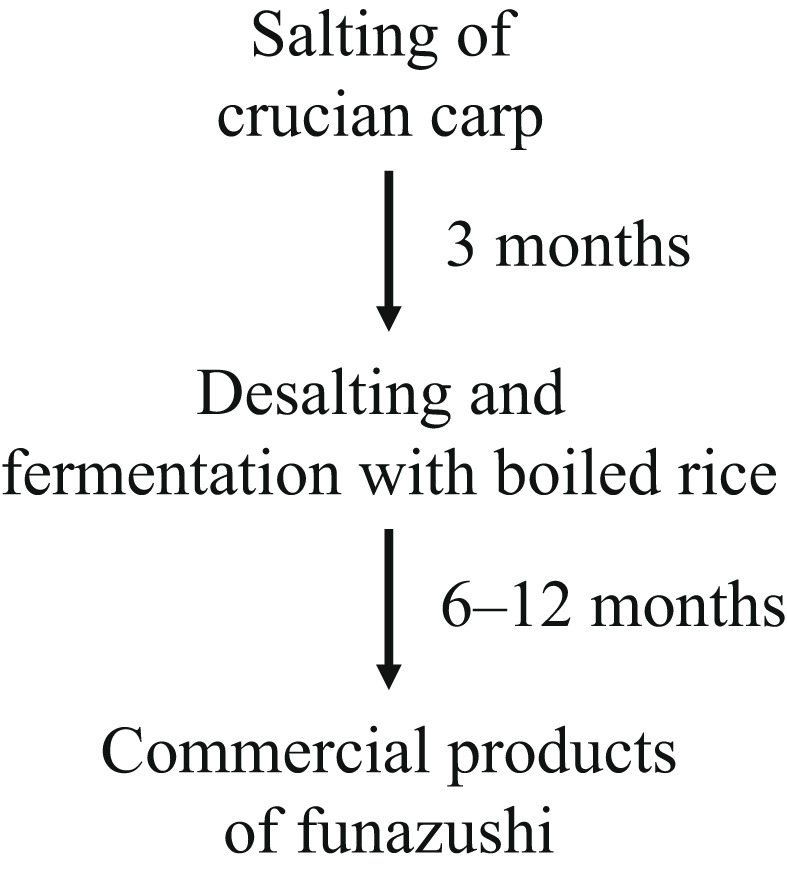
Scheme of the funazushi production process. The point of fermentation with boiled rice was defined as the starting time of fermentation

Funazushi is a unique fermented fish (Figure 1) preparation that is considered as a precursor of modern sushi. For approximately 1500 years (Ito, 2018), it has been made in Shiga prefecture, which contains Lake Biwa, the largest lake in Japan. Crucian carp (funa) and boiled rice are used as ingredients and fermented for more than 6 months to complete the fermentation (Tsuda et al., 2012). Fermentation is a complicated process. The typical funazushi production process is illustrated in Figure 1. Even in recent fermentation processes, starter microbial cultures are not commonly used. It is known that lactic acid bacteria (LABs) are major microbes in fermented commercial products (Tsuda et al., 2012). Isolation of many LAB species during the fermentation process and/or in commercial fermented products has been reported, including *Streptococcus salivarius* (Tsuda et al., 2012), *Companilactobacillus alimentarius* (Santos et al., 2016), *Lactiplantibacillus plantarum* (Tanaka‐Azuma et al., 2009; Tsuda et al., 2012), *Lacticaseibacillus casei* (Tsuda et al., 2012), *Lacticaseibacillus paracasei* (Komatsuzaki et al., 2008), *Ligilactobacillus acidipiscis* (Tsuda et al., 2012), *Companilactobacillus farciminis* (Tsuda et al., 2012), and *Lentilactobacillus buchneri* (Isobe et al., 2002; Tsuda et al., 2012). However, the major LAB species in funazushi products are still controversial.

In traditional fermentation processes, specific microbes dominate after transition even if starter microbial cultures are not used (Gharechahi et al., 2017; Marui et al., 2015; Oshiro et al., 2021). Environmental stress tolerance, the utilization of nutrients such as sugar and nitrogen in a microbial species, and the presence of antimicrobial substances can help microbial species grow solely in fermented foods (domination regarding cell number) (Li et al., 2020; Satora et al., 2020; Zhang et al., 2015). Because funazushi contains 2%–5% (w/v) NaCl and organic acids including 1% (w/v) lactic acid derived from LAB metabolism (Tsuda et al., 2012), tolerance to NaCl and organic acid stress might be critical factors for domination. It is known that many LABs produce bacteriocins, which are antimicrobial peptides effective against related LAB species (Eguchi et al., 2001; Kawamoto et al., 2002). To gain insights into such characteristics of LAB isolates, we employed cultivation‐based methodologies.

In this study, we analyzed major LABs in funazushi produced on the eastern side of Lake Biwa, a main funazushi production area, and the results suggested that *Lentilactobacillus buchneri* dominates in funazushi. The genetic diversity of *L. buchneri* strains was evaluated by amplification of the genes in the CRISPR (clustered regularly interspaced short palindromic repeat) locus. We also described the factors affecting the domination by *L. buchneri*.

## 2 RESULTS

### 2.1 LAB species from funazushi products and intermediate samples collected in the Shiga area of Japan

First, we isolated LABs from funazushi products (nine samples) produced on the eastern side of Lake Biwa in Shiga prefecture, Japan and purchased from grocery stores. LABs were isolated from each sample. In our experimental conditions, LABs were detected if their cell numbers exceeded 1 × 10^2^ cells per gram of sample. The isolated strains were identified according to 16S rDNA sequence homology. The data revealed that all isolates belonged to *L. buchneri* (Table 1), suggesting that the major species in funazushi is *L. buchneri*.

**TABLE 1:** *Lentilactobacillus buchneri* strains isolated from commercial funazushi products

Strain	Source of isolation	Factory	Factory location^a^	Description	Accession number of 16S rDNA
RZ1	Fish	A	Ohmi‐Hachiman		LC660670
RZ2	Fish	A	Ohmi‐Hachiman		LC660671
RZ3	Rice	A	Ohmi‐Hachiman		LC660672
RZ4	Rice	A	Ohmi‐Hachiman		LC660673
RZ5	Fish	A	Ohmi‐Hachiman	Long maturation product	LC660674
RZ6	Rice	A	Ohmi‐Hachiman	Long maturation product	LC660675
RZ7	Fish	A	Ohmi‐Hachiman	Fish cake type^b^	LC660676
RZ8	Fish	A	Ohmi‐Hachiman	Fish cake type^b^	LC660677
RZ9	Fish	B	Ohmi‐Hachiman		LC660678
RZ10	Fish	B	Ohmi‐Hachiman		LC660679
RZ11	Fish	C	Ryuoh		LC660680
AM1	Fish	D	Maibara		LC660681

^a^ City or town located in Shiga prefecture.

^b^ A type of serving in which fish and rice were hashed into small pieces. Funazushi is commonly served as fish meat cut with a thickness of 1 cm.

To gain insights into the LAB species, intermediate samples, including boiled rice and fish, during funazushi fermentation were obtained from funazushi producers. LABs were isolated and identified (Table 2). The data suggested that various LAB species existed in quantities exceeding 1 × 10^2^ cells per gram of sample in the early stage. In the later stage, *L. buchneri* was detected as a major species. Taken together, these results suggest that domination by *L. buchneri* occurred during fermentation.

**TABLE 2:** Microbial strains that appeared at high frequencies during the fermentation of funazushi

Strain	Source of isolation	Time after fermentation (weeks)	Species	Accession number of 16S rDNA
KTL1	Rice	1	*Weissella confusa*	LC656416
KTL2	Rice	1	*Lactiplantibacillus plantarum*	LC656417
KTL3	Rice	1	*Levilactobacillus brevis*	LC656418
KTL4	Rice	2	*Lacticaseibacillus paracasei*	LC656419
KTL5	Rice	2	*Companilactobacillus musae*	LC656420
KTL6	Rice	2	*Lactiplantibacillus plantarum*	LC656421
KTL7	Rice	3	*Lentilactobacillus buchneri*	LC656422
KTL8	Rice	3	*Lactiplantibacillus plantarum*	LC656423
KTL9	Rice	4	*Lentilactobacillus buchneri*	LC656427
KTL10	Rice	4	*Lactiplantibacillus plantarum*	LC656430
KTL11	Rice	8	*Lentilactobacillus buchneri*	LC656433
KTL12	Rice	8	*Lactiplantibacillus plantarum*	LC656432

### 2.2 Genotyping of *L. buchneri* strains isolated from funazushi

**FIGURE 2:**
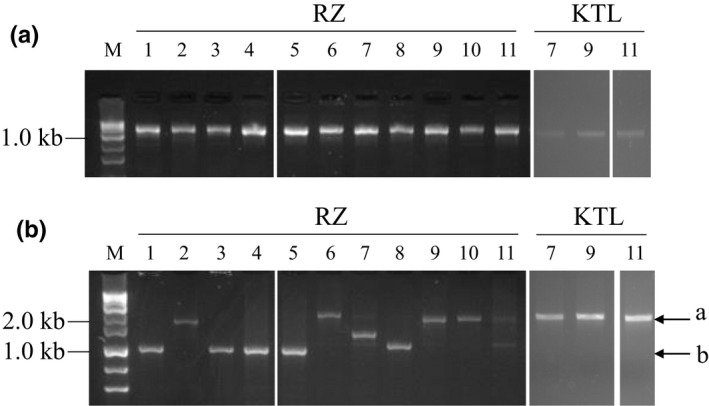
Polymerase chain reaction (PCR)‐based detection of the clustered regularly interspaced short palindromic repeat‐CRISPR‐associated protein (CRISPR‐Cas) elements in *Lentilactobacillus buchneri* strains. Amplification of (a) *cas9* or (b) the hypervariable type II‐A repeat spacer in the CRISPR array in 14 *L. buchneri* isolates by PCR. The strains are listed in Table 1 (RZ1‐11: left side) and 2 (KTL7, 9, and 11: right side). A 0.1–10 kb DNA ladder is presented on the left side of both electrophoresis images

To determine the genetic diversity of the *L. buchneri* isolates, we next performed genotyping analysis as described by Daughtry (Daughtry et al., 2018). *cas9* or the type II‐A repeat spacer at the CRISPR region of each strain was amplified (Figure 2). Polymerase chain reaction (PCR) detected *cas9* in all tested strains (Figure 2a). Amplification of the repeat spacer region resulted in some types of band patterns (Figure 2b). The approximately 2 kb band was observed with RZ2, 9, 10, KTL7, 9, 11 (The position is designated as “a” in Figure 2b). The approximately 1 kb band was detected with RZ1, 3, 4, 5 (The position is designated as “b” in Figure 2b). The rest of the strains exhibited their own characteristic bands with the size distinct from “a” or “b.” These results suggested that several types of *L. buchneri* potentially become dominant species during the fermentation of funazushi.

These results suggest that a funazushi sample contains several genotypes of *L. buchneri* strains. It is speculated that *L. buchneri* strains have critical characteristics promoting their domination in funazushi.

### 2.3 Bacteriocin production abilities of the *L. buchneri* strains

**FIGURE 3:**
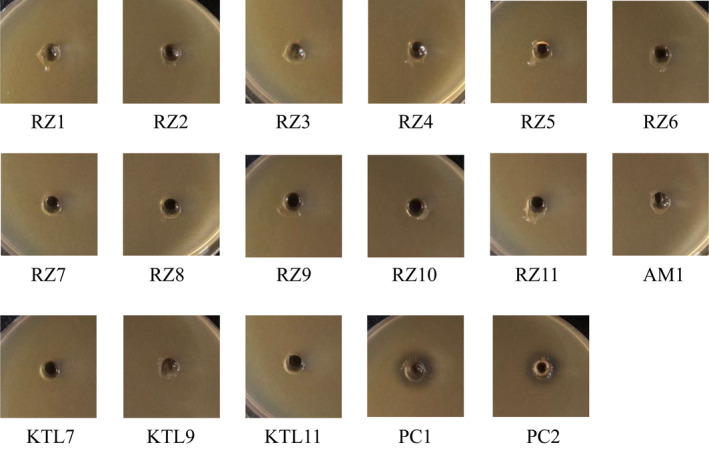
Bacteriocin production abilities of the *Lentilactobacillus buchneri* strains. An aliquot of 20 μl culture supernatants of the isolated *L. buchneri* strains were spotted on an MRS (De Man, Rogosa, and Sharpe) agar plate seeded with *Enterococcus mundtii*. The tested culture supernatant of the strain is indicated below each spotted zone. Upper panels; RZ1‐6, middle panels; RZ7‐10, AM1, and bottom panels; KTL7, 9, 11, PC1, and PC2. PC1 and PC2 indicate the culture supernatants from *Latilactobacillus curvatus* and *Lacticaseibacillus casei*, respectively

To determine the effects of antimicrobial activity on domination, the bacteriocin production abilities of the *L. buchneri* isolates were evaluated. In this assay, we employed *Enterococcus mundtii* NBRC 13712 because it was sensitive to class II bacteriocins (Eguchi et al., 2001). The bacteriocin production abilities of the *L. buchneri* strains presented in Tables 1 and 2 were evaluated. In our experimental conditions, growth inhibition by culture supernatant was not detected in any *L. buchneri* strain (Figure 3). This result suggests that the *L. buchneri* strains from funazushi exhibited little or no bacteriocin production; therefore, it was unlikely that bacteriocins contributed to the microbial domination of *L. buchneri* during funazushi fermentation.

### 2.4 Stress tolerance of the *L. buchneri* strains

**FIGURE 4:**
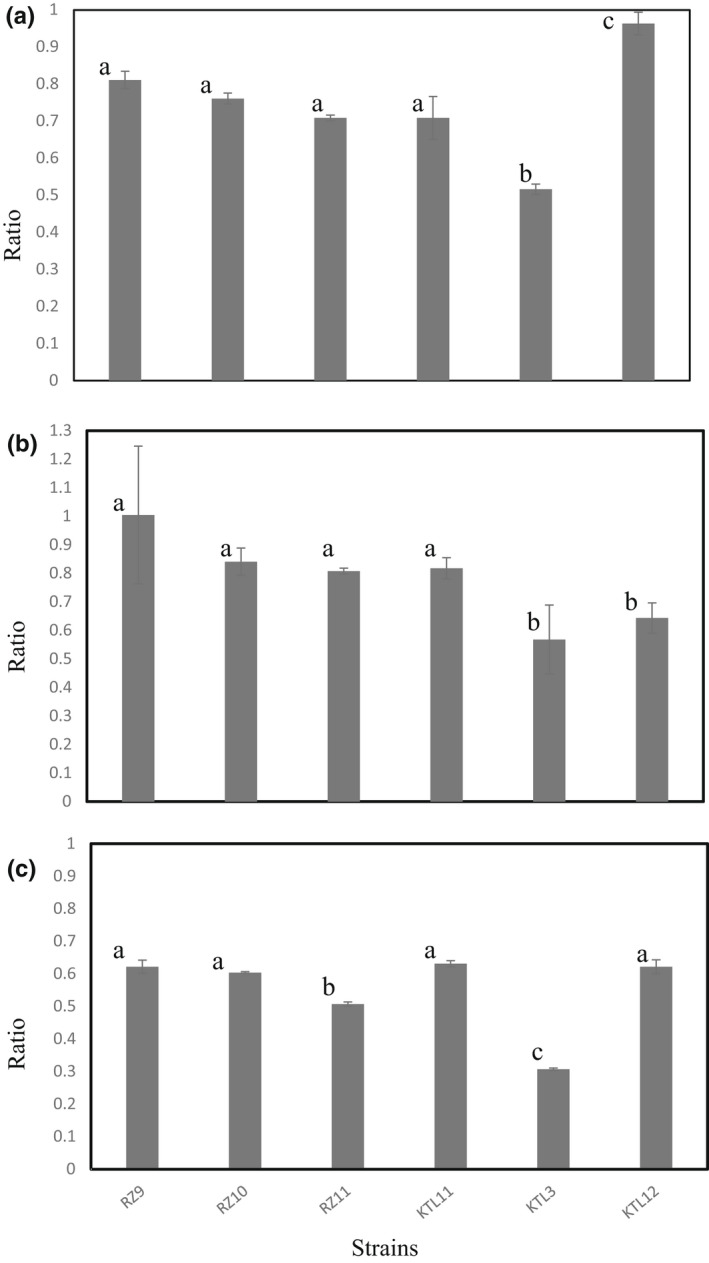
Stress tolerance of *Lentilactobacillus buchneri* under stressed growth conditions. The indicated lactic acid bacterium strains were inoculated in MRS (De Man, Rogosa, and Sharpe) medium containing (a) 0.5 M NaCl, (b) 0.5% lactic acid, or (c) 0.5% acetic acid and anaerobically incubated at 30°C. The optical density at 600 nm of the culture was measured at 24 h. Bars not sharing a common letter are significantly different at *p* < 0.05

To examine the environmental stress tolerance of *L. buchneri*, the environmental stress tolerance of the isolates from commercial funazushi products or fermenting intermediates of funazushi was evaluated. The growth rates of the isolates were compared to those of *Levilactobacillus brevis* KTL3 and *Lactiplantibacillus plantarum* KTL12 (Table 2), which were isolated from fermenting intermediates, under high concentrations of NaCl, lactic acid, and acetic acid (Figure 4). In the presence of 0.5 M NaCl, the growth of the *L. buchneri* strains and the *L. brevis* strain was inhibited by 20%–30% and 48%, respectively (Figure 4a). The *L. plantarum* strain exhibited almost no growth inhibition in the highly salted MRS (De Man, Rogosa, and Sharpe) medium, indicating that *L. buchneri* is not endowed with enhanced salt tolerance. The sensitivity of the *L. buchneri* strains to acetic acid was the same as that of *L. plantarum* (approximately 40% inhibition, Figure 4c). Conversely, it was apparent that the growth of the *L. buchneri* strains was maintained at more than 80% of the control level in the presence of 0.5% lactic acid (Figure 4b). The growth rates of *L. brevis* and *L. plantarum* in the presence of lactic acid were 57% and 64% versus the control, respectively. These results strongly suggest that tolerance to a high concentration (1% (w/v)) of lactic acid in funazushi among other putative stressors contributes to the growth advantage of *L. buchneri* in the later stage of fermentation.

## 3 DISCUSSION

We analyzed LABs in funazushi produced without the addition of microbial starters. LAB species isolated from commercial products differed significantly from those isolated from intermediate samples. This suggested that the domination by *L. buchneri* occurred after the transition of various LAB species. To gain insights into this phenomenon, we employed cultivation strategies and characterized the isolates from funazushi, although recent studies frequently employed culture‐independent strategies for microbial community analysis (Marui et al., 2015; Yap et al., 2021).

Tsuda et al. reported that many LAB species were predominant in funazushi (Tsuda et al., 2012). In our study, *L. buchneri* strains were detected as the dominant species. We consider that this difference might depend on the locations of the production facilities and the maturation stages of the products. We also consider funazushi a suitable model for the analysis of domination by specific microbes during fermentation because the phenomenon of domination is extremely clear. It should be noted that some LABs might not be successfully isolated using our cultivation method. It was expected that the addition of NaCl or lactic acid can improve the isolation of LABs, as LABs in funazushi potentially exhibit enhanced growth in the presence of high concentrations of salt or organic acid.

It was difficult to identify the origins of *L. buchneri* in funazushi. In preliminary experiments, we isolated *Aeromonas*, *Vibrio*, *Pseudomonas*, and *Enterobacter* spp. from the intestinal tract of raw crucian carp. However, *L. buchneri* was not detected in the starter materials including the intestinal tract, gills, or fins of raw crucian carp or boiled rice (data not shown). This implies that the intestine of crucian carp was not the origin of these bacteria. Possible origins of *L. buchneri* are workspace environments and containers such as barrels.

Genotyping of the CRISPR locus in *L. buchneri* strains demonstrated that the strains isolated from funazushi have distinct differences, which were also observed in the length of the repeat spacer locus among strains. It was intriguing that the genotypes of strains differed both between strains and within single samples. These results indicate that in the final fermentation step of funazushi, *L. buchneri* consists of several strains rather than a single strain. Taken together, the results suggest that *L. buchneri* strains may share unique characteristics, such as environmental stress tolerance, for domination in fermented foods.

To determine the factors associated with domination, we have evaluated bacteriocin production and environmental stress tolerance in *L. buchneri* isolates. Although *L. buchneri* was reported to produce a bacteriocin (buchnericin) (Yildirim et al., 2002), our isolates did not produce the antibacterial substances. Several researchers reported that bacteriocins play significant roles in microbial domination in fermented foods (Janssen et al., 2018; Plessas et al., 2011). We therefore think that antimicrobial substances do not influence bacterial domination during fermentation of funazushi. Conversely, we found that *L. buchneri* strains have high stress tolerance to lactic acid. It is reported that LABs are damaged by lactate, one of their own metabolites. It is possible that the tolerance to the main metabolite produced by LABs helps *L. buchneri* become the dominant species during extremely long incubation (6 or more months in the preparation of funazushi). In addition, *L. buchneri* is known to produce acetic acid together with lactic acid under both aerobic and anaerobic conditions (Heinl et al., 2012; Heinl & Grabherr, 2017). This species can also metabolize lactic acid to acetic acid and 1,2‐propanediol under anaerobic and acidic conditions (Oude Elferink et al., 2001). These characteristics of the metabolism of *L. buchneri* resulted in higher viability than observed for other bacterial species, and they may also help this species dominate the microbial community of funazushi during long‐term incubation. Several metabolites other than lactic acid have been detected in funazushi (Mukai‐Kubo et al., 2007); therefore, the tolerance to unknown metabolites produced during fermentation may also support *L. buchneri* becoming the dominant species. Metabolomic analysis of funazushi will help to clarify the process by which *L. buchneri* becomes the dominant species in fermented food.

The biota of funazushi, especially during fermentation, has not been intensively examined. In this study, transition and domination during funazushi fermentation were investigated for the first time. Cultivation‐based analysis of *L. buchneri* and other LAB strains enabled our finding of the robust growth property of *L. buchneri*. The results of the present study indicated that the environmental stress tolerance of LABs might be a critical characteristic for domination and survival in the environments of fermenting foods.

Recently, many researchers have taken an interest in interactions and competition among microbes in the environments of fermented foods using omics‐based analyses (18). The results of the present cultivation‐based study indicate that environmental stress tolerance is an important factor for domination in fermented foods.

## 4 MATERIALS AND METHODS

### 4.1 Medium and growth conditions

The MRS agar medium (Becton, Dickinson and Company, Franklin Lakes, NJ, USA) was used for the growth and maintenance of LABs. Cultivation was performed at 30°C under anaerobic conditions prepared using Aneropak Kenki (Sugiyama‐Gen Co., Ltd., Tokyo, Japan), unless otherwise stated.

### 4.2 Funazushi samples

Commercial funazushi products were purchased from grocery stores located on the eastern side of Lake Biwa in Shiga prefecture, Japan. Fermenting samples were provided by a funazushi company in Hikone City, Shiga prefecture.

### 4.3 Isolation of LAB

Samples (approximately 0.5 g) of a commercial funazushi or fermenting intermediates were separated into fish and boiled rice and suspended in 5 ml of saline. Portions (100 μl) of the suspension were plated on MRS agar medium. After 48 h of incubation, morphologically distinct bacterial colonies were selected. The isolates were purified twice using MRS agar medium. The isolated microbial strains were preserved at −80°C.

### 4.4 Taxonomic identification

Taxonomic identification of the isolated strains was based on a partial sequence of 16S rDNA (primer pairs: 341F and 806R) using a previously described method (Kawamoto et al., 2002). The primers for amplifying and sequencing 16S rDNA (primer pairs: 341F and 806R) were described elsewhere (16S metagenomic sequencing library preparation Part# 15044223 Rev. B). We determined the homology of the sequences by conducting a BLAST (Basic Local Alignment Search Tool) search of the DNA Data Bank of Japan (DDBJ). The partial 16S rDNA sequences of the LAB strains obtained in this study were deposited with the DDBJ. The accession numbers of the rDNA sequences are presented in Tables 1 and 2. LAB strains with greater than 99.5% homology with *L. buchneri* DSM 20057^T^ were identified as *L. buchneri*.

### 4.5 Genetic analysis of the CRISPR region of *L. buchneri* strains

Polymerase chain reaction (PCR) with designated primers (Briner & Barrangou, 2014) was performed using crude genomic DNA (gDNA) fractions prepared by InstaGene™ Matrix (Bio‐Rad Laboratories) following the manufacturer's instructions. The gDNA samples for each strain were extracted from two independent bacterial cultures, and the reproducible results were obtained with each sample. Quick‐Load Purple 1 kb Plus DNA Ladder (New England Biolabs) was used as a DNA size marker.

### 4.6 Bacteriocin assay

The spot‐on‐lawn method as described by van Reenen et al. was used to determine antimicrobial activity (van Reenen et al., 1998). Briefly, the isolated *L. buchneri* strains were cultured overnight in 5 ml MRS medium. An aliquot of 20 μl culture supernatants of the *L. buchneri* were spotted on an MRS agar plate seeded with overnight culture of the test bacterial strain (*Enterococcus mundtii* NBRC13712) at 0.25% (v/v). The culture supernatants of two LAB strains, a *Latilactobacillus curvatus* and a *Lacticaseibacillus casei*, were employed as positive controls. They were isolated from the local pickles in Japan and confirmed to produce clear growth‐inhibitory zone in our previous examination. The growth‐inhibitory zones around the culture supernatants of *L. curvatus* and the *L. casei* were approximately 8 mm and 7 mm in diameter, respectively.

### 4.7 Environmental stress tolerance assay

The LAB cells with an identical optical density (OD, 0.5) were inoculated in 5 ml of MRS broth containing 0.5 M NaCl, 0.5% lactic acid, or 0.5% acetic acid and anaerobically incubated at 30°C. The OD at 600 nm (OD_600_) was measured at 24 h. The ratio of OD_600_ relative to that with no stressor (control) was indicated. The mean and SD of OD_600_ were calculated from triplicate results.

### 4.8 Statistical analysis

Values were expressed as the mean ± SD. Differences in means between groups were tested by the Tukey–Kramer test using the js‐STAR program (http://www.kisnet.or.jp/nappa/software/star8/info/new.htm). A *p*‐value of less than 0.05 was considered statistically significant.

## CONFLICT OF INTEREST

The authors declare that they do not have any conflict of interest.

## Supporting information


Figure S1

## Data Availability

Data derived from public domain resources
