# Supplementary material for: Lentilactobacillus buchneri domination during the fermentation of Japanese traditional fermented fish (funazushi)
Source: Food Sci Nutr. 2022 Jul 27;10(11):4073–9. doi: 10.1002/fsn3.3002 (PMC9632191; doi:10.1002/fsn3.3002)
Supplement: Supplementary file 1 — Figure S1 [file FSN3-10-4073-s001.pptx]

## Slide 1
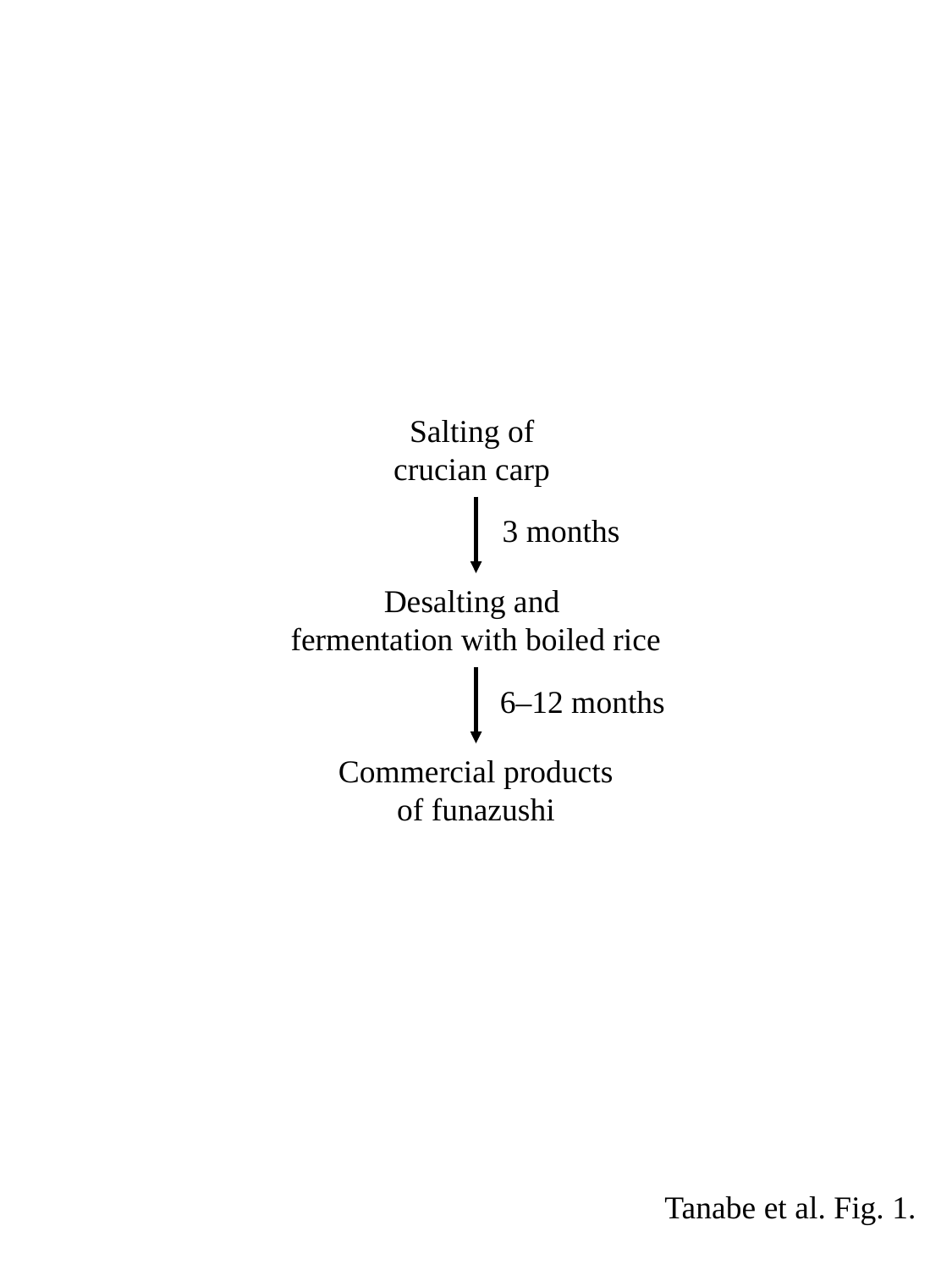

Salting of
crucian carp
3 months
Desalting and
fermentation with boiled rice
6–12 months
Commercial products
of funazushi
Tanabe et al. Fig. 1.

## Slide 2
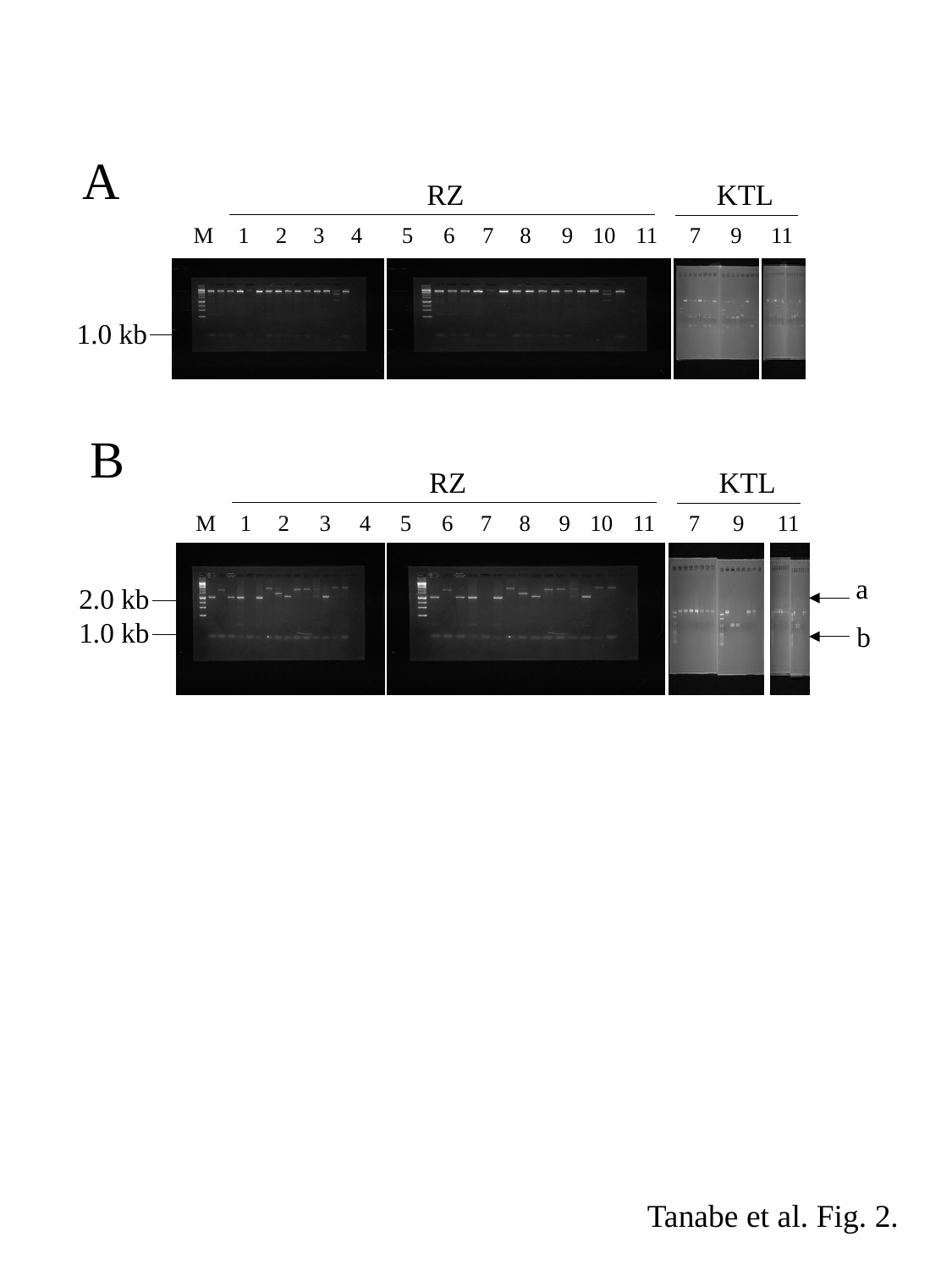

A
RZ
KTL
M
1
2
3
4
5
6
7
8
9
10
11
7
9
11
1.0 kb
B
RZ
KTL
M
1
2
3
4
5
6
7
8
9
10
11
7
9
11
a
2.0 kb
1.0 kb
b
Tanabe et al. Fig. 2.

## Slide 3
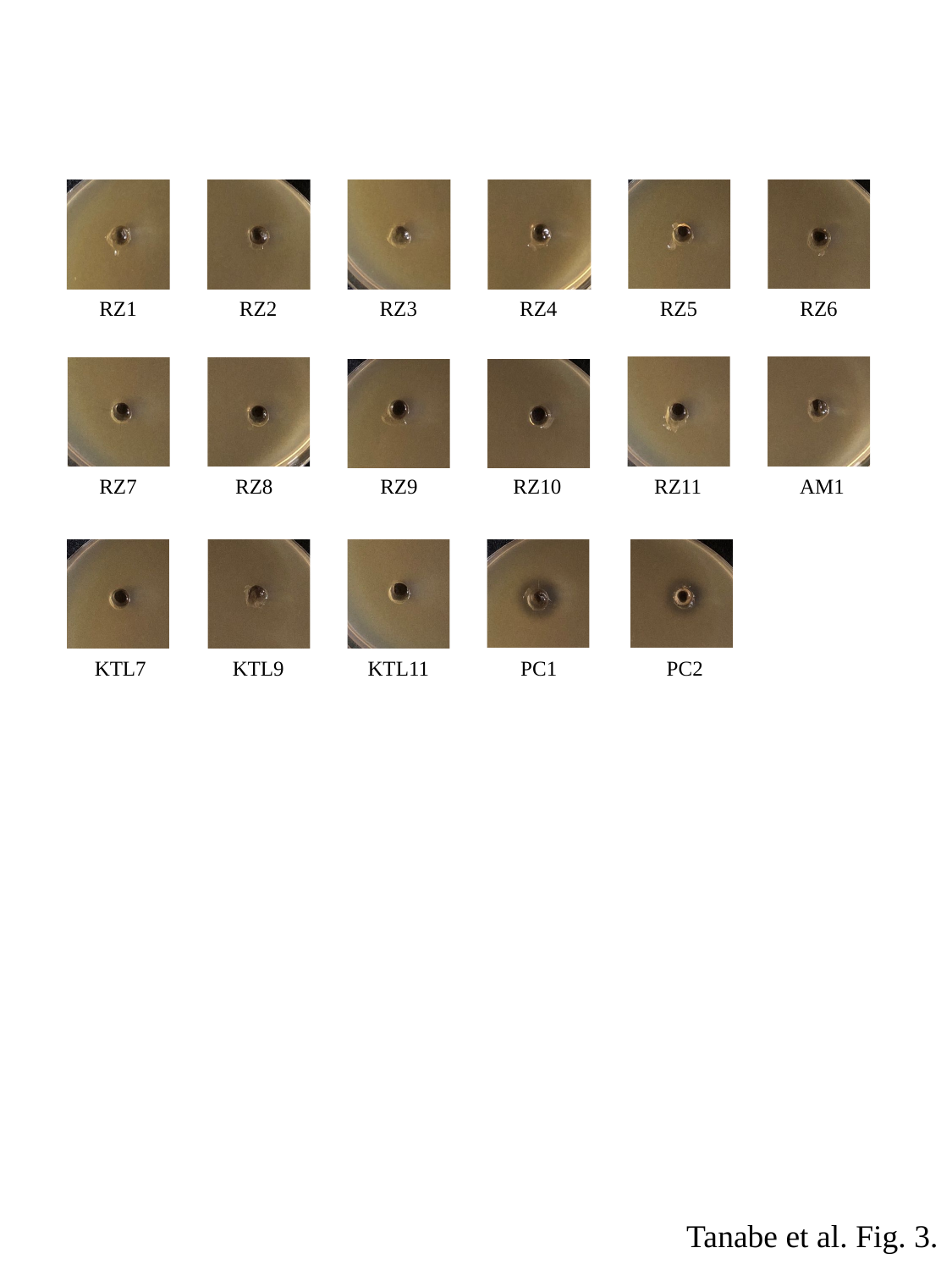

RZ1
RZ2
RZ3
RZ4
RZ5
RZ6
RZ7
RZ8
RZ9
RZ10
RZ11
AM1
KTL7
KTL9
KTL11
PC1
PC2
Tanabe et al. Fig. 3.

## Slide 4
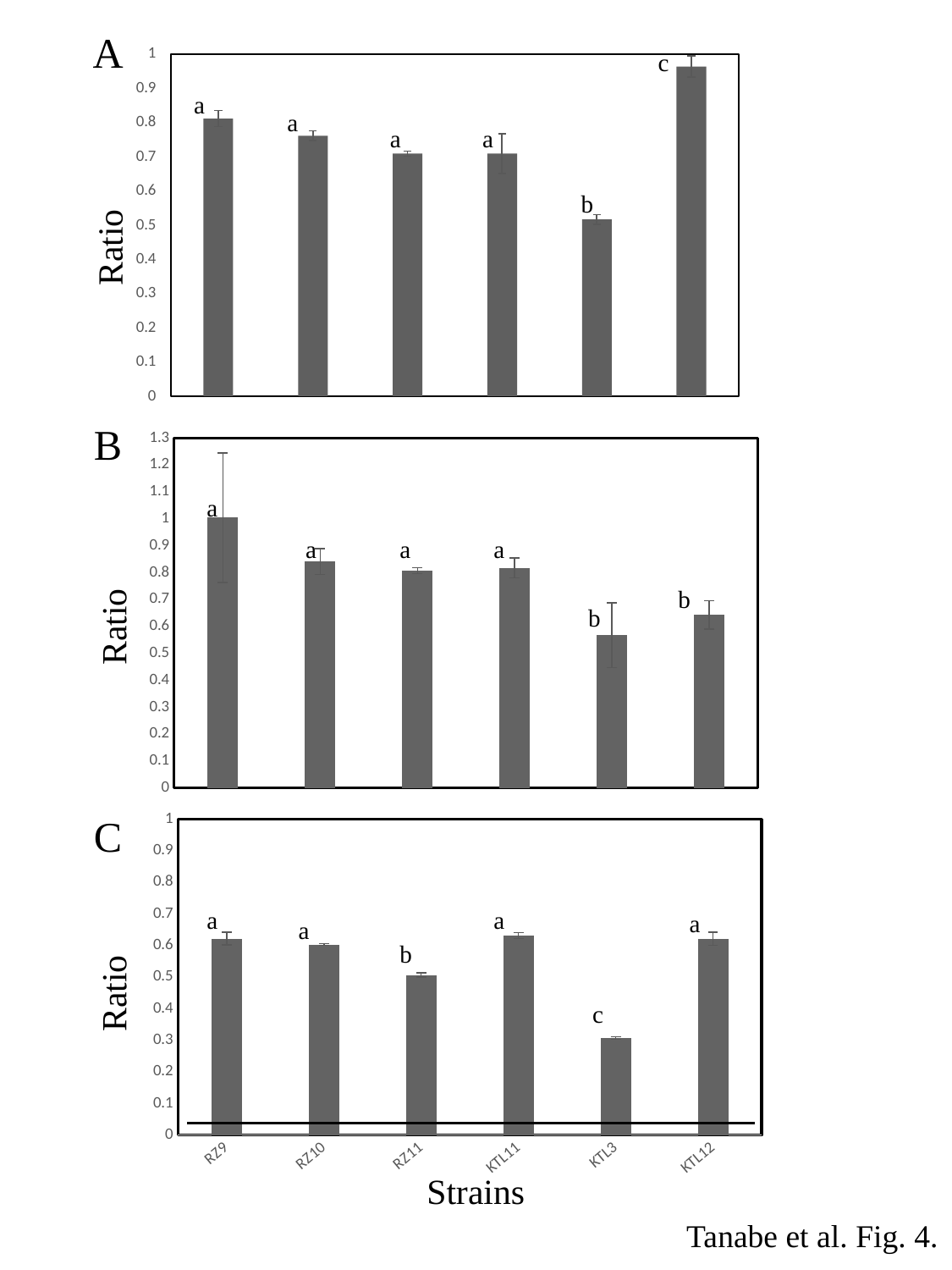

A
c
a
a
a
a
b
Ratio
B
### Chart
| Category | ％ |
|---|---|
| RZ9 | 1.0048363095238095 |
| RZ10 | 0.8411993337034979 |
| RZ11 | 0.807982740021575 |
| KTL11 | 0.8179979777553085 |
| KTL3 | 0.568193008370261 |
| KTL12 | 0.6435340022296544 |a
a
a
a
b
b
Ratio
C
### Chart
| Category | ％ |
|---|---|
| RZ9 | 0.6220238095238096 |
| RZ10 | 0.6035535813436979 |
| RZ11 | 0.5070118662351673 |
| KTL11 | 0.6314459049544996 |
| KTL3 | 0.3072378138847858 |
| KTL12 | 0.6220735785953178 |a
a
a
a
b
Ratio
c
Strains
Tanabe et al. Fig. 4.

## Slide 5
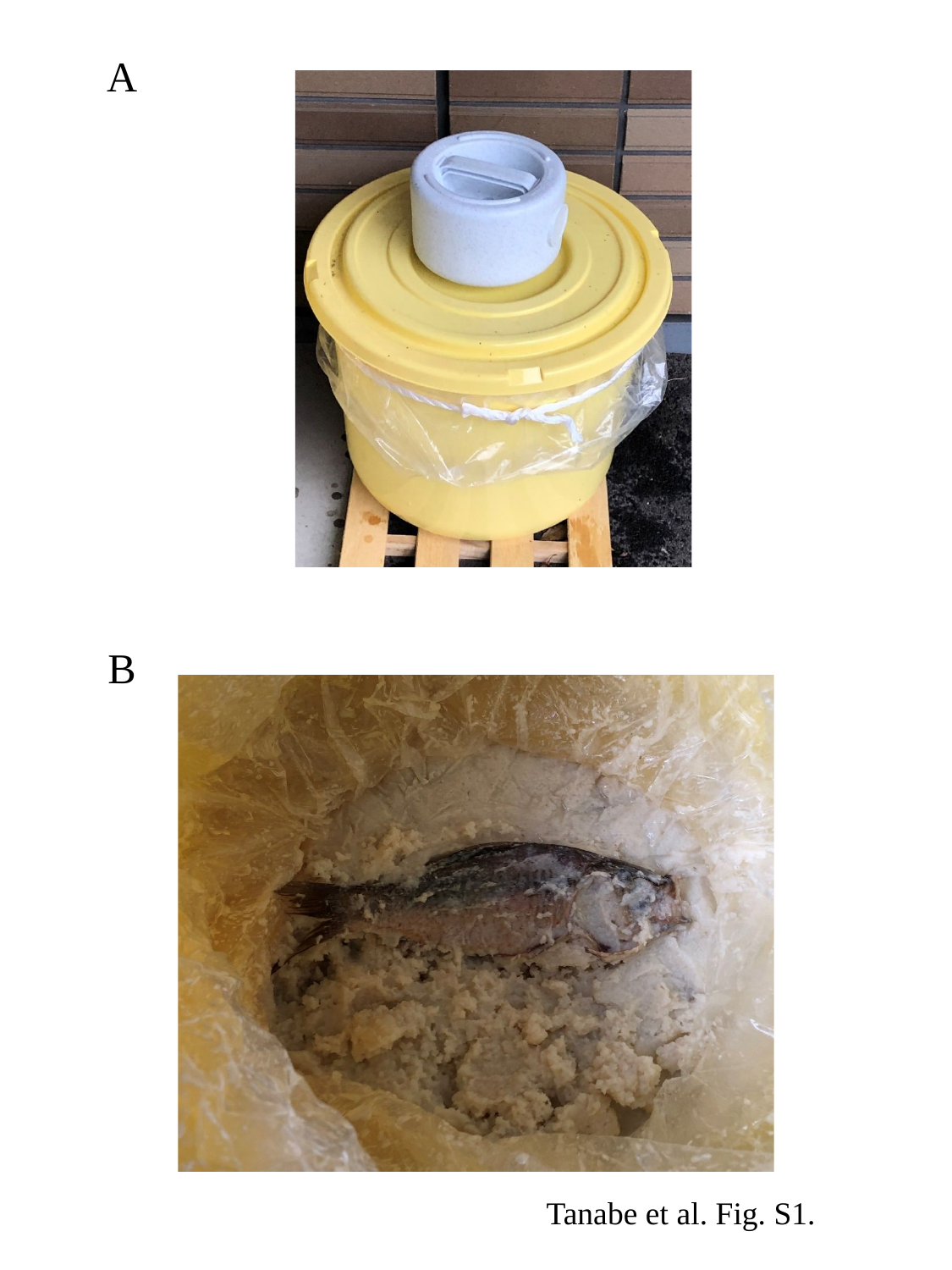

A
B
Tanabe et al. Fig. S1.
